# Health-related quality of life of HIV patients with comorbidities of hypertension or diabetes in Ghana

**DOI:** 10.3389/fpubh.2024.1383743

**Published:** 2024-11-19

**Authors:** Richmond Owusu, Emmanuel Bugyei Kwarteng, Serwaa Akoto Bawua, Desmond Dzidzornu Otoo, Justice Nonvignon

**Affiliations:** ^1^Department of Health Policy, Planning and Management, School of Public Health, University of Ghana, Accra, Ghana; ^2^Department of Biological, Environmental, and Occupational Health, School of Public Health, University of Ghana, Accra, Ghana

**Keywords:** HIV, health-related quality of life, Ghana, interventions, hypertension, diabetes, EQ-5D-5L

## Abstract

**Background:**

Clinical studies, particularly in the context of HIV/AIDS, have utilized health-related quality of life (HRQoL) and health state utility values to assess both clinical and economic implications. Improvement in HIV management with antiretroviral therapy (ART) has coincided with an increased morbidity of chronic conditions such as diabetes and hypertension among people living with HIV. The study offers empirical evidence establishing a link between HIV, comorbidities like hypertension and diabetes, and their collective impact on HRQoL.

**Methods:**

A cross-sectional quantitative study among 418 HIV patients in three regions of Ghana in the Savannah, Middle Belt, and Coastal belt. Face-to-face interviews were conducted using EuroQol-5 Dimensions-5 Levels (EQ-5D-5L) in combination with EuroQol-Visual Analog Scales (EQ-VAS). Ugandan EQ-5D-5L value set was used for computing the utility values. Patients' socio-demographic and clinical data were obtained from medical records and analyzed descriptively. Mann-Whitney U and Kruskal Wallis tests were used to evaluate the relationship between patient socio-demographic and clinical variables and health status.

**Results:**

Overall median utility value was 0.93(IQR=0.79, 1.00) from the EQ-5D index and 90 (IQR = 80, 100) from the EQ-VAS scores. Prevalence of hypertension and diabetes were 9.1% and 2.4% respectively. There was significant difference in EQ-VAS score between HIV patients with hypertension and those without hypertension (z = 2.146 *p* = 0.032). There was also a significant difference in the EQ-VAS scores (z = 2.381, *p* = 0.017) of patients with diabetes and those without diabetes. Region, sex, age, education, marital status, employment status, place of residence, monthly income level, duration of infection, adherence to ART, and other illness were significantly associated with EQ-5D-5L utility and EQ-VAS scores at 0.05 significant level.

**Conclusion:**

HRQoL among HIV positive individuals in Ghana was high. Comorbidities such as hypertension or diabetes had nuanced effects on perceived health status. A patient-centered, multidisciplinary approach to HIV care should be adopted considering regional differences and the presence of comorbidities.

## Introduction

The concept of Quality of Life (QoL) which refers to an individual's total wellbeing is intricate, encompassing various contexts and influenced by factors such as personal perspectives, social interactions, environment, and both physical and mental health (1). Several studies have highlighted the impact of clinical considerations, cultural and personal values, as well as social context on the quality of life (1, 2). In situations where quality of life is considered in relation to health and illness, it is sometimes referred to as health-related quality of life (HRQoL) to distinguish it from other dimensions of quality of life such as social and environmental quality of life (3). HRQoL incorporates elements associated with the physical domains of the individual, in addition to factors pertaining to the individual's mental and social dimensions (4). When evaluating treatments for Human Immunodeficiency Virus (HIV) or acquired immunodeficiency syndrome (AIDS), both clinical and economic assessments are conducted, utilizing measures such as patient HRQoL and health state utility values (5). These evaluations provide a comprehensive understanding of the effectiveness of treatments, considering not only clinical outcomes but also the broader impact on individuals' wellbeing in various aspects of their lives.

The World Health Organization reports that HIV remains a serious global public health problem. It reported a death toll of about 42.3 million individuals with certain countries witnessing an alarming surge in newly reported cases following a period of steady decline (6). According to the Joint United Nations Programme on HIV/AIDS (UNAIDS), more people than ever now have advanced HIV infection, which is a factor in the global rise in AIDS prevalence (7). Availability of antiretroviral therapy (ART) has allowed people with HIV to live longer with a growing number of individuals aging with the virus. Hypertension and diabetes, two common chronic comorbidities, have gained prominence in this population since older individuals with HIV are at higher risks of developing comorbid conditions such as hypertension and diabetes (8, 9). These conditions, if not effectively managed, can lead to severe complications, including cardiovascular diseases, kidney disorders, and neurologic impairments (10, 11). The coexistence of HIV with hypertension and/or diabetes poses a multifaceted healthcare challenge, demanding an in-depth exploration of its impact on the overall wellbeing of affected individuals.

Sub-Saharan Africa (SSA) contributes to over 65% of the people living with HIV (PLHIV) (12). While countries in SSA grapple with infectious diseases such as HIV, Malaria, and Tuberculosis, there has been increasing incidence of non-communicable diseases (NCDs) (13). The most prevalent non-communicable diseases (NCDs) in Ghana include cardiovascular disease, cancer, chronic respiratory disorders, diabetes, and sickle cell disease (14). Studies report that cardiovascular diseases and diabetes contribute the largest national NCD burden in Ghana (15–17). NCDs account for about 65% of all fatalities in Ghana, and over 100,000 premature deaths and 10,500 lost of disability-adjusted life years (DALYs) for every 100,000 people in Ghana (15, 18).

In Ghana, the number of people with hypertensive heart disease and diabetes has increased significantly and features in the top 10 causes of death in all health facilities yearly (19). Ghana is experiencing epidemiological and demographic transition which means the incidence of NCDs will continue to rise in the coming years. Non-Communicable Diseases are anticipated to become the leading cause of death by 2030 in SSA including Ghana (14).

Many studies have evaluated the HRQoL of individuals living with HIV in several countries including Ethiopia (5), Columbia (20), and Nigeria (8). In Ethiopia, it was found that individuals living with HIV exhibit high HRQoL with median EQ-5D index and the EQ-VAS scores of 0.94 and 80 respectively. However, issues such as anxiety/depression and pain/discomfort were prevalent (5). EQ-5D-5L scores of 0.85 ± 0.21 and EQ-VAS score of 84 ± 14 were reported in Bogotá, Colombia among HIV patients receiving antiretroviral therapy (20). Although these studies reported high HRQoL, most of them were limited to solitary healthcare settings and comorbidities.

Despite the importance of QoL measurement and its usefulness in improving health outcomes and cumulative impact, it remains largely under-researched particularly in low and middle-income countries (LMICs). Notably, within the Ghanaian context, there is a dearth of studies assessing the HRQoL among patients dealing with the co-morbidities of HIV and conditions such as hypertension or diabetes.

This study seeks to address this gap by specifically investigating the HRQoL among individuals in Ghana who are simultaneously managing HIV and contending with the challenges of coexisting hypertension or diabetes. By focusing on this intersection of health concerns, the research aims to contribute valuable insights into the HRQoL of a population facing complex health conditions, ultimately fostering a better understanding of their unique needs and experiences.

## Methods

### Study design and setting

The study utilized a cross-sectional design with a quantitative approach to assess the HRQoL among HIV patients and investigate the prevalence of hypertension and diabetes. Cross-sectional studies offer a momentary depiction of outcomes and associated attributes at a specific point in time, making them suitable for capturing individual characteristics and desired outcomes concurrently (21). This design facilitated the examination of the relationship between individual characteristics and the targeted health outcomes. The study was conducted in three regions of Ghana, spanning the Savannah, middle belt, and coastal belt ecological zones, specifically in the Greater Accra, Ashanti, and Upper West regions. These regions were purposively selected due to the high prevalence of HIV cases in their respective ecological zones, as reported in the latest Ghana AIDS Commission fact sheets from 2019 (22). ART clinics in Madina Polyclinic, Kumasi South Hospital, and Wa Municipal Hospital were selected as survey sites. These ART clinics are well established healthcare facilities that provide antiretroviral therapy and other essential services like chronic care. Additionally, these facilities are situated within the capital of the selected regions and therefore have a large population of HIV patients and also serve as a referral point for ART services for other facilities within their respective regions.

### Study population, sample size and sampling

The study population comprised individuals 18 years and above living with HIV/AIDS who visited the HIV treatment clinic at the selected facilities from August to September 2023. The sample size for this study was determined using the Cochran formula (23). The determination of the sample size for the study was derived from the prevalence of hypertension in individuals who are HIV positive. Considering hypertension being the most prevalent comorbidity of 30.8% as compared to diabetes (24). A convenience sampling technique was employed to select HIV patients who were eligible to partake in the study. Participants were recruited at the health facility level; thus, HIV treatment centers were purposively selected for recruiting potential respondents. PLHIV who were present and accessible at the HIV treatment center on each day of data collection during clinic hours (8am−4pm) were selected and each participant was given a unique identification code. Those individuals who met the stipulated criteria for inclusion and provided written informed consent were chosen to participate in the study until the minimum sample size was achieved. Given the sensitive nature of HIV and the significant stigma associated, respondents were selected based on their availability and willingness to participate at the time of data collection.

#### Data collection

The study employed the EuroQoL 5-dimension instrument (EQ-5D) to measure HRQoL, utilizing the 5-level EQ-5D (EQ-5D-5L) and the EuroQoL-visual analog scale (EQ-VAS). EQ-5D-5L, designed with five dimensions assessing mobility, self-care, usual activities, pain/discomfort, and anxiety/depression across five severity levels, allowed for a comprehensive evaluation of participants' health states. The VAS recorded overall health on a 20 cm vertical scale that features endpoints denoted as 0 (“the worst health you can imagine”) and 100 (“the best health you can imagine”) (25). The EQ-5D-5L has demonstrated good measurement properties, including reliability, validity, and responsiveness, when used in HIV/AIDS populations across various settings (26, 27). Additionally, as a preference-based instrument, the EQ-5D-5L provides utility scores that can be used for economic evaluations and cost-utility analyses (26, 28). The questionnaire which was administered in English and local dialects, collected socio-demographic and clinical characteristics data from medical records after obtaining written informed consent from respondents. Respondents self-reported the presence of hypertension and diabetes. Additionally, clinical characteristics data obtained from the medical records included the disease duration, duration on ART, CD4 count, blood pressure, viral load and other illnesses. Adherence to ART was self-reported by the respondents. A structured questionnaire with close-ended questions facilitated face-to-face data collection, administered by trained health workers who served as research assistants in about 10–15-min interviews. Data was collected from August to September 2023 with monthly income reflecting the 2023 currency year.

#### Data processing and analysis

Data collected was entered into Microsoft Excel 2016 by two research assistants, checked and cleaned by the researcher and then imported into STATA version 16 for analysis. Descriptive statistics such as frequency, percentage, mean, median, and standard deviation were used to present socio-demographic and clinical data, as well as EQ-5D-5L health profiles, EQ-VAS, and EQ-5D utility scores. The EQ-5D-5L value sets for Uganda were employed for the valuation of participants' health states due to the absence of value sets for Ghana at the time. Also, Uganda's cultural, socio-economic, and health system characteristics are similar to that of Ghana and offers a close approximation of HRQoL valuations. These similarities suggest that the health preferences and quality of life valuations of individuals from both countries might be more aligned. The value sets were applied by converting 5-digit health states to single summary index values using corresponding weights (29). HRQoL scores were determined, and normality tests revealed non-normal distribution for EQ-5D-5L utility and EQ-VAS scores. Non-parametric statistical analyses, including Mann-Whitney U and Kruskal Wallis tests, were utilized to assess associations between patient variables and health status (30). The outcome variables were EQ-5D utility scores and EQ-VAS scores. Independent variables included socio-demographic characteristics and respondents' clinical data (viral load, blood pressure, other illnesses). *P*-values <0.05 were considered statistically significant.

### Ethical considerations

Ethical clearance was obtained from the Ghana Health Service Ethics Review Committee with reference number: GHS-ERC027/05/23. Written informed consent was obtained from all respondents before data collection.

## Results

### Participants' demographic and clinical characteristics

The study enrolled a total of 418 patients with wide-ranging demographic and clinical characteristics. From Table 1, majority of respondents 55.2% (*n* = 231) come from the Greater Accra region, followed by Ashanti 32.8% (*n* = 137) and Upper West 12.0% (*n* = 50). The patient population was predominantly female 82.3% (*n* = 344). The median age of the participants was 40 (IQR = 33, 49) years. The majority of the participants, 32.3% (*n* = 135), fell within the age group of 30–39 years.

**Table 1:** Patient socio-demographic and clinical characteristics.

**Variable**	**Frequency**	**Percent (%)**
**Region:**		
Greater Accra	231	55.2
Ashanti	137	32.8
Upper West	50	12.0
**Sex:**		
Male	74	17.7
Female	344	82.3
**Age (years):**		
18–29	64	15.3
30–39	135	32.3
40–49	117	28.0
50–59	69	16.5
≥ 60	33	7.9
Median (IQR)	40 (33, 49)	
**Education:**		
None	65	15.6
Primary/JHS	200	47.9
Secondary	119	28.5
Tertiary	34	8.1
**Marital status:**		
Never married	86	20.6
Married	168	40.2
Co-habiting	45	10.8
Widowed	53	12.7
Divorced	66	15.8
**Current employment status:**		
Employed/Self-employed	342	81.8
Unemployed	68	16.3
Student	8	1.9
**Place of residence:**		
Rural	33	7.9
Urban	385	92.1
**Monthly income level in 2023 (GHS):**		
<1,000	293	70.1
1,000–2,000	66	15.8
>2,000	19	5.5
Do not receive income	40	9.6
**Duration of infection**		
10 yrs or less	354	84.7
>10 yrs	64	15.3
Median (IQR)	4 (3, 9)	
**Duration on ART in months**		
<12	84	20.1
12–48	128	30.6
>48	206	49.3
**Adherence to ART**		
Adherence	400	95.7
Non-adherence	18	4.3
**Viral load:**		
Undetected (<40 copies/mL)	318	76.1
Detected (≥40 copies/mL)	100	23.9
**Blood pressure:**		
Controlled (<140/90 mm Hg)	326	78.0
Not Controlled (≥140/90 mm Hg)	92	22.0
**Other illness:**		
Asthma	7	1.7
Blindness	1	0.2
Boil/Swollen arm	1	0.2
Cripple	1	0.2
Dental caries	1	0.2
Epilepsy	2	0.5
Fibroid	1	0.2
Hemorrhoids	1	0.2
Hearing impairment	1	0.2
Hepatitis B	5	1.2
Hepatitis C	1	0.2
Hernia	1	0.2
Rheumatic	1	0.2
Stroke	2	0.5
Typhoid fever	1	0.2
Ulcer	11	2.6
No other illness	380	90.9

JHS, Junior high school.

Nearly half of the participants (47.9%, *n* = 200) had completed only Primary/JHS education, while just 8.1% (*n* = 34) had attained tertiary education. Also, the majority (40.2%, *n* = 168) of participants were married. The majority of respondents (81.8%, *n* = 342) were either employed or self-employed, whereas only 1.9% (*n* = 8) were students. Additionally, 92.1% (*n* = 385) of participants lived in urban areas. Furthermore, 70.1% (*n* = 293) had a monthly income less than GHS1,000, only 5.5% (*n* = 19) had monthly income more than GHS2,000 and 9.6% (*n* = 40) reported no monthly income. The median duration of HIV infection was 4 (IQR=3, 9) years with 84.7% (*n* = 354) of respondents reported to have been infected for 10 years or less. Moreover, almost half (49.3%, *n* = 206) of respondents reported being on ART for more than 48 months. The majority of respondents (95.7%, *n* = 400) were adherent to ART. Also, the majority (76.1%, *n* = 318) of participants had an undetected viral load and the majority had controlled blood pressure (78%, *n* = 326). In addition, 9.1% (*n* = 38) reported having other illnesses, with ulcers being the most reported illness followed by asthma.

### Self-reported health profile of people living with HIV

From Table 2, it can be observed that a considerable majority of the participants, specifically 73.9% (*n* = 309) had no problem with mobility. Additionally, the majority of respondents 93.5% (*n* = 391) reported no difficulties with self-care. The majority of respondents (84.7% *n* = 354) had no problems with regular activities. In relation to pain and discomfort, 66.0% (*n* = 276) reported no problems. A considerable percentage reported slight to extreme pain or discomfort (34%). More than half of respondents 53.4% (*n* = 223) reported not being worried or depressed. However, almost half (46.7%) reported slight to extreme levels of worry and depression.

**Table 2:** Frequency of self-reported health profile of HIV/AIDS patients.

**Health Profile**	**Mobility (walking) *n* (%)**	**Self-care *n* (%)**	**Regular activities *n* (%)**	**Pain/discomfort *n* (%)**	**Worries (anxiety)/depression *n* (%)**
No problems	309 (73.9)	391 (93.5)	354 (84.7)	276 (66.0)	223 (53.4)
Slight problems	68 (16.3)	18 (4.3)	46 (11.0)	82 (19.6)	114 (27.3)
Moderate problems	20 (4.8)	4 (1.0)	10 (2.3)	31 (7.4)	30 (7.2)
Severe problems	19 (4.6)	3 (0.7)	4 (1.0)	17 (4.1)	25 (6.0)
Unable/extreme problems	2 (0.5)	2 (0.5)	4 (1.0)	12 (2.9)	26 (6.2)

### Health-related quality of life score for people living with HIV

Table 3 indicates the health status indices for the total sample along with the values stratified by age. The median EQ-VAS score was 90.00 (IQR = 80.00, 100.00) and the median EQ-5D utility score was 0.93 (IQR=0.79, 1.00). Respondents within the age group of 18–29 years reported the highest EQ-VAS score and EQ-5D utility score of 87.27± 14.11 and 0.88± 0.24 respectively. On the other hand, participants within the age group ≥60 years reported the lowest mean EQ-VAS score and EQ-5D utility score of 79.39± 15.09 and 0.69± 0.41 respectively.

**Table 3:** EQ-5D-5L and EQ-VAS score of PLHIV by age groups (years).

**Health status index**	**Age (years)**					
	**18-29**	**30-39**	**40-49**	**50-59**	≥**60**	**Total**
EQ-VAS score; mean (SD)	87.27 (14.11)	86.70 (15.35)	85.34 (17.12)	83.28 (17.65)	79.39 (15.09)	85.26 (16.14)
Median	90.00	90.00	90.00	90.00	80.00	90.00
25^th^ percentile	80.00	80.00	80.00	70.00	70.00	80.00
75^th^ percentile	100.00	100.00	100.00	100.00	90.00	100.00
**EQ-5D utility**						
Mean (SD)	0.88 (0.24)	0.86 (0.23)	0.82 (0.28)	0.75 (0.36)	0.69 (0.41)	0.82 (0.29)
Median	0.95	0.95	0.92	0.87	0.80	0.93
25th percentile	0.87	0.85	0.74	0.72	0.65	0.79
75th percentile	1	1	1	1	0.95	1

### Prevalence of hypertension and diabetes among HIV patients

From Table 4, the prevalence of hypertension was 9.1%. The median EQ-5D utility score was 0.87 (IQR = 0.77, 0.95) for hypertensive patients and 0.94 (IQR = 0.78, 1.00) for non-hypertensive patients. The Mann-Whitney U test shows a test statistic of z=1.755 with a *p*-value of 0.079, indicating that there is no statistically significant difference in EQ-5D utility scores between the two groups. However, the median EQ-VAS score was 85 (IQR = 70, 90) for hypertensive patients and 90 (IQR = 80, 100) for non-hypertensive patients. The Mann-Whitney U test shows a test statistic of z=2.146 with a *p*-value of 0.032, which is significant at *p* < 0.05.

**Table 4:** HRQoL of HIV patients with hypertension and diabetes.

**Variable**			**EQ-5D utility**			**EQ-VAS score**	
	***N*** **(%)**	**Median (IQR)**	**Test statistic**	***P*** **-value**	**Median (IQR)**	**Test statistic**	***P*** **-value**
Hypertensive	38(9.1)	0.87(0.77, 0.95)	1.755	0.079	85(70, 90)	2.146	**0.032**
Non-Hypertensive	380(90.9)	0.94(0.78, 1.00)			90(80, 100)		
Diabetic	10(2.4)	0.86(0.72, 1.00)	0.680	0.497	80(60, 87)	2.381	**0.017**
Non-diabetic	408(97.6)	0.93(0.79, 1.00)			90(80, 100)		

Mann-Whitney U test. Bold figures are significant at *p* < 0.05.

On the other hand, the prevalence of diabetes was 2.4%. The median EQ-5D utility score was 0.86 (IQR = 0.72, 1.00) for diabetic patients and 0.93 (IQR = 0.79, 1.00) for non-diabetic patients. The Mann-Whitney U test shows a test statistic of z = 0.680 with a *p*-value of 0.497, indicating that there is no statistically significant difference in EQ-5D utility scores between the two groups. However, the median EQ-VAS score was 80 (IQR = 60, 87) for diabetic patients and 90 (80,100) for non-diabetic patients. The Mann-Whitney U test shows a test statistic of z = 2.381 with a *p*-value of 0.017, which is statistically significant at *p* < 0.05.

### Factors influencing HIV patients' HRQoL

Table 5 reports the association of patients' characteristics with HRQoL. From the Kruskal Wallis test, there was a significant difference in EQ-5D utility score [χ2_(2)_ =19.696, *p* < 0.001] among the three regions. No significant difference in EQ-VAS scores was observed among regions. The Mann-Whitney U test showed no significant difference in EQ-5D utility score between male and female, however, there was a significant difference in EQ-VAS scores (z = 2.836, *p* = 0.005). There was a significant difference in both EQ-5D utility [χ2_(2)_ =17.357, *p* = 0.002] and EQ-VAS score [χ2_(2)_ =9.679, *p* = 0.046] among age groups. Scores tend to decrease with increasing age, with the ≥60 age group displaying the lowest median score. Moreover, educational levels significantly influence EQ-5D utility scores [χ2_(2)_ = 11.139, *p* = 0.011]. Tertiary-educated individuals reported higher EQ-5D utility scores compared to those with lower educational levels.

**Table 5:** Influence of patient variables on health status (N = 418).

**Variable**	**EQ-5D utility**				**EQ-VAS score**			
	**Median (IQR)**	**Mean rank**	**Test statistic**	***P*** **-value**	**Median (IQR)**	**Mean rank**	**Test statistic**	***P*** **-value**
**Region** ^b^			19.696	**<0.001**			1.736	0.420
Greater Accra	0.95 (0.86,1.00)	232			90 (80,100)	214		
Ashanti	0.86 (0.72,1.00)	184			90 (75,100)	198		
Upper West	0.87 (0.66,0.95)	172			90 (85,98)	216		
**Sex** ^a^			−1.530	0.126			2.836	**0.005**
Male	0.95 (0.85,1.00)	229			85 (70,95)	174		
Female	0.92 (0.77,1.00)	205			90 (80,100)	217		
**Age (years)** ^b^			17.357	**0.002**			9.679	**0.046**
18–29	0.95 (0.87,1.00)	239			90 (80,100)	221		
30–39	0.95 (0.85,1.00)	226			90 (80,100)	219		
40–49	0.92 (0.74,1.00)	202			90 (80,100)	213		
50–59	0.87 (0.72,1.00)	191			90 (70,100)	198		
≥60	0.80 (0.65,0.95)	150			80 (70,90)	153		
**Education** ^b^			11.139	**0.011**			3.006	0.391
None	0.87 (0.76,0.95)	178			85 (80,98)	190		
Primary/JHS	0.92 (0.74,1.00)	204			90 (80,100)	215		
Secondary	0.95 (0.82,1.00)	223			90 (80,100)	213		
Tertiary	0.98 (0.87,1.00)	253			90 (80,95)	193		
**Marital status** ^b^			18.826	**0.001**			3.939	0.414
Never married	0.95 (0.85,1.00)	225			90 (80,100)	209		
Married	0.93 (0.79,1.00)	213			90 (80,100)	219		
Co-habiting	1.00 (0.82,1.00)	251			90 (80,100)	214		
Widowed	0.85 (0.66,0.95)	156			85 (70,99)	185		
Divorced	0.88 (0.76,1.00)	196			90 (75,100)	200		
**Employment status** ^b^			14.322	**0.001**			0.506	0.776
Employed/Self-employed	0.94 (0.80,1.00)	218			90 (80,100)	211		
Unemployed	0.81 (0.59,0.95)	161			90 (77.5,98)	200		
Student	0.98 (0.82,1.00)	250			90 (70,100)	212		
**Place of residence** ^a^			−2.732	**0.006**			−0.644	0.520
Rural	0.80 (0.55,0.95)	155			87 (70,100)	196		
Urban	0.94 (0.79,1.00)	214			90 (80,100)	210		
**Monthly income level (GHS)** ^b^			7.988	**0.046**			0.928	0.819
<1,000	0.92 (0.79,1.00)	207			90 (80,100)	211		
1,000–2,000	0.95 (0.81,1.00)	231			90 (75,100)	200		
>2,000	0.95 (0.89,1.00)	246			90 (80,100)	225		
No income	0.86 (0.65,0.98)	173			90 (80,98)	201		
**Duration of infection** ^a^			2.959	**0.003**			0.530	0.596
10 yrs and less	0.94 (0.79,1.00)	217			90 (80,100)	210		
More than 10 yrs	0.84 (0.66,0.98)	169			87.5 (75,100)	202		
**Duration on ART in months** ^b^			2.977	0.226			3.503	0.174
<12	0.90 (0.78,0.98)	196			90 (80,95)	196		
12–48	0.95 (0.79,1.00)	223			90 (80,100)	225		
More than 48	0.93 (0.77,1.00)	207			90 (78,100)	205		
**Adherence to ART** ^a^			−2.297	**0.022**			−1.781	0.075
Adherence	0.93 (0.79,1.00)	212			90 (80,100)	211		
Non-adherence	0.79 (0.61,0.94)	147			82.5 (70,90)	160		
**Viral load** ^a^			−1.191	0.234			1.521	0.128
Undetected (<40 copies/mL)	0.94 (0.79,1.00)	213			90 (80,100)	204		
Detected (≥40 copies/mL)	0.92 (0.77,1.00)	197			90 (80,100)	225		
**Blood pressure** ^a^			1.172	0.241			0.944	0.345
Controlled (<140/90mm Hg)	0.93 (0.79,1.00)	213			90 (80,100)	212		
Not Controlled (≥140/90 mm Hg)	0.92 (0.75,1.00)	197			90 (80,99)	197		
**Other illness** ^a^			2.899	**0.004**			2.271	**0.023**
Reported illness	0.81 (0.57,0.95)	215			82.5 (70,95)	167		
No illness	0.94 (0.79,1.00)	156			90 (80,100)	213		

^a^Mann-Whitney U test. ^b^Kruskal Wallis test. Bold figures are significant at *p* < 0.05.

Also, there was a significant difference in EQ-5D utility scores [χ2_(2)_ = 18.826, p = 0.001] among the marital status categories with those co-habiting reporting the highest EQ-D utility scores. Additionally, there was a significant difference in the EQ-5D utility scores [χ2_(2)_ = 14.322, *p* = 0.001] among the employment status categories but no difference in the EQ-VAS score. Students reported higher EQ-5D utility scores compared to other employment categories. A significant difference was found in the EQ-5D utility score (z = −2.732, *p* = 0.006) between the two categories of place of residence, but no significant difference was observed in the EQ-VAS score. Urban residents reported higher EQ-5D utility scores compared to rural residents. Moreover, there was a marginally significant difference in the EQ-5D utility score [χ2_(2)_ = 7.988, *p* = 0.046] across the various monthly income levels. Duration of infection significantly influences EQ-5D utility scores (z = 2.959, *p* = 0.003). Individuals with longer infection durations (>10 years) tend to have slightly lower EQ-5D utility scores. Furthermore, there was a significant difference in the EQ-5D utility scores among patients' adherence level, with those adhering to ART tending to have higher EQ-5D utility scores compared to non-adhering individuals. There is a significant difference in both the EQ-5D utility score (z = 2.899, *p* = 0.004) and EQ-VAS score (z = 2.271, *p* = 0.023) between HIV patients who reported other illness and patients who did not report any illness.

## Discussion

The study included a diverse group of 418 HIV patients with representation across different ecological zones, this signifies a heterogeneous patient population. The majority of the participants were female, constituting 82.3% of the sample. This sex distribution reflects the global trend where women are disproportionately affected by HIV (31). In sub-Saharan Africa, about 62% of new HIV infections were among females of all ages. This corroborates the findings of this study. However, in some other jurisdictions, males of all ages experienced a proportionately higher incidence of HIV infections as of 2023 (31). The median age of the participants was 40 years, with a wide age range from 18 to 76 years. The self-reported health profiles of HIV/AIDS patients indicate varying degrees of challenges across different dimensions of health.

The prevalent challenges reported by respondents predominantly centered around the pain/discomfort and anxiety/depression dimensions of the EQ-5D-5L. Consistent with prior research conducted in Ethiopia, Nigeria, and Colombia, individuals living with HIV/AIDS exhibited a higher frequency of issues related to the anxiety/depression dimension compared to the general population (5, 8, 20). This underscores the necessity for an elevated standard of healthcare provision tailored to mitigate the health challenges faced by HIV/AIDS patients, with a particular emphasis on mental and palliative care. It is crucial to recognize that even mild levels of anxiety or depression can significantly impact an individual's overall wellbeing and quality of life, emphasizing the importance of addressing these concerns and offering appropriate psychological support.

The study's findings reveal a relatively high level of self-assessed HRQoL among HIV patients. Similar to a study conducted by Osei-Yeboal et al. (32) in the Volta region of Ghana, approximately 89% of PLHIV reported high HRQoL (32). This could be due to improvement in the access to ART and other support services, improvement in awareness and education, adequate psychosocial support including counseling and mental health resources, government initiatives and efforts by stakeholders, especially the non-governmental organizations (NGOs) to address HIV/AIDS. Although these scores align with those reported in Ethiopia (5) and England (33), they are comparatively lower than the findings of studies by Keaei et al. (20) in Colombia and Popping et al. (33) in the Netherlands. Additionally, it was observed that the perceived health status and overall quality of life of PLHIV tend to decline with increasing age. This underscores the significance of recognizing age-related disparities in healthcare planning and interventions. Consequently, acknowledging these differences becomes essential for the development of targeted interventions that focus on enhancing wellbeing and addressing the distinct needs of various age cohorts within the PLHIV community.

Only 9.1% of the survey respondents acknowledged having hypertension, a notably low prevalence considering that hypertension is a common comorbidity in individuals with HIV (34). In a prior study conducted in Ghana, the reported prevalence of hypertension was significantly higher at 30.8% (24). The unexpectedly lower prevalence observed in our study may be attributed to various factors, including underreporting of lack of awareness regarding one's health conditions. Also, the prevalence of diabetes among PLHIV was only 2.4%. This finding aligns with results from a study in Nigeria (8) and is lower than previous reports in Ghana (35, 36). Similar to hypertension, the surprisingly low prevalence of diabetes is noteworthy, given the increased risk of diabetes in HIV patients (37). This lower prevalence might be linked to underreporting or lack of awareness among the affected individuals. Alternatively, due to the high adherence rates to ART, HIV patients often engage in frequent interactions with healthcare personnel during routine follow-up appointments. This consistent contact facilitates the provision of education and counseling regarding the prevention of additional health conditions, such as NCDs, which may contribute to the observed lower prevalence rates of these conditions within this population. These outcomes emphasize the crucial need for regular health screenings, monitoring, and education for individuals with HIV who have comorbidities. The results suggest that a portion of the population may have undiagnosed or unreported hypertension and diabetes. Effective management of these comorbid conditions is paramount for ensuring the overall wellbeing of individuals living with HIV. The relatively low prevalence of these comorbidities also prompts further research to gain a deeper understanding of the factors contributing to their rarity in this specific population.

The study revealed a potential association between hypertension and a diminished HRQoL among HIV patients. While the difference in EQ-5D utility scores does not reach statistical significance, the EQ-VAS scores portray a meaningful disparity. Hypertensive individuals demonstrated lower median EQ-VAS scores, suggesting a potential impact on their perceived health and wellbeing compared to non-hypertensive peers. In the case of diabetes, though the difference in EQ-5D utility scores isn't statistically significant, a notable discrepancy emerges in EQ-VAS scores between diabetic and non-diabetic HIV patients. This discrepancy signifies a potential impact of diabetes on the subjective health perception of individuals, indicating a lower perceived health status among diabetic individuals.

These findings hold significance in clinical settings. Addressing hypertension and diabetes management among HIV patients might not only target the management of these conditions but also potentially improve their perceived health and overall wellbeing. It emphasizes the need for a holistic approach in healthcare interventions, considering not only the primary condition (HIV) but also comorbidities and their effects on the individual's HRQoL (38).

This study also delved into various patient-related factors that contribute to the perceived health status among individuals with HIV. Notably, regional disparities were observed, with significantly higher EQ-5D utility and EQ-VAS scores in Greater Accra compared to Ashanti and Upper West regions. These differences may indicate potential health disparities influenced by variations in healthcare infrastructure, accessibility, or socio-economic factors across these regions (39–41).

Moreover, gender emerged as a factor influencing perceived health status, as reflected in the EQ-VAS scores. While EQ-5D utility did not show gender disparity, females exhibited notably higher EQ-VAS scores. The reasons for this gender-based difference are likely multifaceted, involving biological, psychological, and sociocultural factors. This result aligns with the findings of Melaku et al. (42) but contrasts with studies conducted in Nigeria (8) and Ethiopia (5). Understanding these variations contributes to a more nuanced comprehension of how different factors may impact the perceived health status of HIV patients in diverse contexts.

Age emerges as a significant factor affecting health perception, with younger age groups reporting better health status compared to their older counterparts. This phenomenon may be attributed to the slower progression of diseases among younger individuals, bolstered immune systems, and robust social networks, including supportive family and friends. These networks contribute emotional and practical support, thereby positively impacting mental health and overall quality of life. This discovery corroborates with prior research findings (42, 43) and contrast with the conclusions drawn in Jackson et al. (8). Additionally, this underscores the imperative for age-specific interventions and support systems tailored to address the unique needs of older adult individuals living with HIV.

Educational attainment and employment status demonstrate a positive correlation with higher perceived health. Individuals with tertiary education, those employed or self-employed, and those with a monthly income exceeding GHS 1,000 tend to exhibit better health perception. This underscores the potential role of socio-economic empowerment in fostering improved health outcomes among individuals living with HIV, as evidenced by existing studies (39–41, 44). Other socio-demographic factors, including marital status and residence, were also identified as significant influences on the HRQoL of PLHIV. Understanding and addressing these factors are crucial in formulating comprehensive strategies to enhance the wellbeing of individuals by navigating the complexities of living with HIV.

The results derived from this study underscore the impact of infection duration on patients' HRQoL. Notably, individuals living with HIV for a period of 10 years or less reported higher HRQoL compared to those with more than 10 years of living with the virus. This disparity may be attributed to the progression of the disease over time, with prolonged exposure potentially leading to advanced stages and consequential effects on health status.

Moreover, the study revealed a significant correlation between adherence to ART and EQ-5D utility scores. This implies that individuals who adhere more closely to their antiretroviral treatment exhibit a higher HRQoL. These findings align with previous studies that have reported similar positive associations (43, 45, 46). The efficacy of ART is evident in its ability to suppress HIV replication, resulting in a substantial reduction in AIDS-related mortality, morbidity, and symptomatic experiences among PLHIV (1, 47, 48).

Additionally, a noteworthy discrepancy in HRQoL was observed among individuals living with HIV who disclosed the presence of other comorbidities in comparison to those who did not. This disparity suggests that the existence of additional illnesses is linked to a diminished state of HRQoL. Consistent with these findings, the study by Van Duin et al. (49) also highlighted a substantial association between the presence of multiple comorbidities and utility scores (49). This emphasizes the intricate relationship between the overall health status of individuals with HIV and the presence of concurrent health challenges, further highlighting the importance of a comprehensive approach to healthcare for this population.

## Conclusion

The study revealed improved quality of life among HIV-positive individuals in Ghana. It underscores the effect of demographic, clinical, and socioeconomic factors on quality of life. However, regional differences significantly influenced health perceptions among HIV patients, with Greater Accra showing higher health scores compared to the Ashanti and Upper West regions. The significant disparities in HRQoL between regions and across different income levels highlight the need for targeted interventions to address these disparities. Additionally, the findings indicated that only a small proportion of these individuals reported coexisting hypertension or diabetes. Interestingly, hypertension and diabetes appeared to have nuanced effects on the perceived health status of individuals living with HIV. The higher HRQoL scores among individuals who are adherent to ART emphasize the importance of sustained treatment and adherence in managing HIV effectively.

In light of these observations, the study underscores the significance of adopting a patient-centered, multidisciplinary approach to HIV care. Recognizing the regional variations and the impact of co-morbidities on health perceptions emphasizes the need for tailored and comprehensive strategies to address the diverse needs of HIV-positive individuals, contributing to an improved quality of life and overall wellbeing. Future studies should focus on longitudinal studies to provide a comprehensive understanding and develop causal relationships to lay foundations for more effective policies and interventions.

## Limitations and strengths

This study reports a few limitations that were observed. First, the study's cross-sectional nature might limit the ability to establish causality. Also, due to the low level of education of most of the HIV patients, it was necessary for skilled staff at the ART unit of the facilities to assist in comprehending the study questionnaire, originally intended to be self-administered. This might have introduced some biases in the responses. Lastly, there might be biases as patients self-reported their hypertension and diabetic status. The confinement of the EQ-5D-5L to 5 dimensions and 5 levels can limit the ability to capture subtle changes in health status. The EQ-VAS may be subject to subjective biases or may not be highly sensitive to detect small changes in health.

However, these tools have been widely used and validated for various populations. This study provided a robust and comprehensive assessment of HRQoL among HIV patients by conducting the study in three regions (Greater Accra, Ashanti, and Upper West) across three distinct ecological zones in Ghana. Thus, it offers insights into regional disparities in HRQoL, enhancing the generalizability of the findings within the country.

## Data Availability

The original contributions presented in the study are included in the article/supplementary material, further inquiries can be directed to the corresponding author.
